# Metabolomics in clinical and forensic toxicology, sports anti‐doping and veterinary residues

**DOI:** 10.1002/dta.3245

**Published:** 2022-03-08

**Authors:** Bethany Keen, Adam Cawley, Brian Reedy, Shanlin Fu

**Affiliations:** ^1^ Centre for Forensic Science University of Technology Sydney Broadway New South Wales Australia; ^2^ Australian Racing Forensic Laboratory Racing NSW Sydney New South Wales Australia; ^3^ School of Mathematical and Physical Sciences University of Technology Sydney Broadway New South Wales Australia

**Keywords:** anti‐doping, equine, metabolomics, toxicology, veterinary residues

## Abstract

Metabolomics is a multidisciplinary field providing workflows for complementary approaches to conventional analytical determinations. It allows for the study of metabolically related groups of compounds or even the study of novel pathways within the biological system. The procedural stages of metabolomics; experimental design, sample preparation, analytical determinations, data processing and statistical analysis, compound identification and validation strategies are explored in this review. The selected approach will depend on the type of study being conducted. Experimental design influences the whole metabolomics workflow and thus needs to be properly assessed to ensure sufficient sample size, minimal introduced and biological variation and appropriate statistical power. Sample preparation needs to be simple, yet potentially global in order to detect as many compounds as possible. Analytical determinations need to be optimised either for the list of targeted compounds or a universal approach. Data processing and statistical analysis approaches vary widely and need to be better harmonised for review and interpretation. This includes validation strategies that are currently deficient in many presented workflows. Common compound identification approaches have been explored in this review. Metabolomics applications are discussed for clinical and forensic toxicology, human and equine sports anti‐doping and veterinary residues.

## Graphical abstract

Metabolomics is a multidisciplinary field that requires knowledge of biology, chemistry and statistics. The metabolomics workflow, encompassing experimental design, sample preparation, analytical determinations, data processing and statistical analysis, requires harmonisation for effective translation. This review covers the stages of the metabolomics workflow, validation strategies and current applications in clinical and forensic toxicology, human anti‐doping, equine research and veterinary residues.

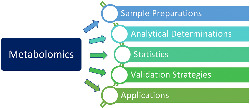

## 1 INTRODUCTION

Metabolomics was first introduced as *metabonomics* and generally focused on comparison between control and diseased samples, for drug safety purposes.^1^ Metabonomics measured a multi‐cellular or organism response to a stimulus whereas metabolomics focuses on measuring a specific cell type or tissue for metabolites secreted by the sample type or found within it.^1^, ^2^, ^3^, ^4^ Metabolomics has moved the focus from conventional studies of a single set of compounds to a network of compounds and metabolites to understand the dynamic multiparametric response of a living system to stimuli.^4^, ^5^, ^6^ Metabolomics has been considered the ideal “omics” technique as it provides a more direct reading of metabolic activities which can be related to a phenotype.^7^, ^8^, ^9^ The metabolome consists of compounds, including but not limited to organic compounds such as amino acids and nucleotides.^10^, ^11^ Metabolomics studies these small molecules (e.g., <2 kDa) using a multivariate approach within biological samples to identify biomarkers.^12^, ^13^ Metabolomics can have a targeted (focusing on a specific group of compounds or metabolic pathway) and/or an untargeted (where an unrestricted number of compounds are monitored) approach. Teale et al. define a biomarker as “any measurable parameter altered as a result of a challenge to an individual's system.”^14^ This can enable investigations into responses from specific stimuli that would otherwise require multiple individual assays to assess the metabolites affected.^12^, ^15^

Metabolomics has evolved over the past 20 years as a multidisciplinary area that includes drug discovery and development.^11^, ^16^, ^17^ It commonly employs the use of nuclear magnetic resonance (NMR) and/or mass spectrometric (MS) data to measure the effects of stimuli.^11^, ^15^, ^18^, ^19^ The implementation of these techniques into routine settings can aid the identification of specific metabolic changes and ultimately lead to a greater understanding of processes in different fields of science, such as physiology and toxicology.^9^ Therefore, a metabolomics approach has the potential to provide a complementary analysis framework in human and equine anti‐doping.^20^, ^21^ Common metabolomic workflows follow a chronological order of experimental design, sample collection and preparation, analytical determinations, statistical analysis and compound identification.^11^ This review will explore each of these stages, together with validation strategies, and discuss selected applications that highlight the benefits of a metabolomic approach.

## 2 EXPERIMENTAL DESIGN

Metabolomics, unlike traditional analytical approaches, has many aspects to consider during the experimental design process. For example, whether a targeted or untargeted metabolomics approach is going to be used will define the future aspects of the workflow. Pre‐analysis considerations may include the instrument selection, column chemistries and ionisation techniques.

The experimental design needs to account for variation; whether this be introduced or biological variance.^22^ Introduced variance may be attributed to sample preparation, analytical determinations and/or data processing and statistical analysis.^23^ Biological variance is commonly seen within metabolomics studies^23^, ^24^ due to gender, age, circadian rhythm and environmental factors. Factors of stress, excessive exercise, disorders involving growth and/or hormones are known to affect the steroidal profile.^25^ Therefore, reasonably large population studies are required to evaluate the variance. A complementary approach to account for biological variance is the introduction of an endogenous reference compound (ERC). ERCs, which are metabolically related or chemically similar to the target compound, can be used as a comparative tool to measure change. Progestins, corticosteroids and other adrenal precursors are often used as ERCs for hormone‐related studies. The ERC can provide an internal normalisation with the hypothesis that it remains stable for the experiment period. It follows that a biomarker ratio can also be established using the biomarker and ERC unaffected by the stimuli being investigated.^26^ Another approach is to explore mixed‐effects modelling in the data‐processing pipeline. This was demonstrated by Wanichthanarak et al. using previously published clinical metabolomics data, thus resulting in a better classification model.^24^

Metabolomic effects, due to treatment, may be small and difficult to detect such as correlated metabolites within a known pathway or uncorrelated metabolites in an unknown pathway. Effects may also display a delayed response to the treatment or varied scale of the response. Therefore, the statistical power of the experiment needs to be considered when planning the sample size for a study.^22^

Data analysis methods (i.e., univariate or multivariate) should also be considered as part of experimental design, not post‐acquisition of results. The suitability of parametric or non‐parametric statistical tests will be influenced by the sample size of the study.

### 2.1 Targeted and untargeted metabolomics

Targeted metabolomics aims to obtain information from, and quantify the presence of a pre‐defined set of compounds. Information relating to compounds of interest is required prior to the investigation.^12^ Targeted metabolomics is a common approach for nutrition research.^27^ However, this is not classified as a true “omics” approach as it is limited in analyte scope.^10^ Many direct detection methods only target parent compounds, which is not always useful.^28^, ^29^ This is particularly notable in environmental studies, when the metabolites of the parent compounds are generally more toxic.^28^ However, it needs to be considered that the bioactivity of some drugs may last longer than the detection periods for the parent drug itself.^30^

An untargeted approach can potentially reduce bias when screening for all metabolites in a sample and the resulting “signature” can then be used to identify novel biomarkers that are associated with a particular physiological state.^10^ This is considered to be a true “omics” approach.^19^ Entities found from untargeted MS analyses are often described in terms of their mass‐to‐charge (*m/z*) values and the intensity of detected ions.^31^ For MS‐based methods, the number of metabolites detected in an untargeted approach is dependent on the sample preparation, column chemistry and ionisation techniques used. Untargeted metabolomics is not limited to a pre‐defined list of compounds and aims to detect anything that is significantly changed in the metabolome. Untargeted analysis results in compounds which can be identified as potential diagnostic tools (biomarkers) for which a targeted method can then be developed.^10^ High mass accuracy may be required to elucidate structures.^21^ Workflows for untargeted metabolomics can be considered indirect detection strategies that measure the effect of substance administration or exposure. Although untargeted metabolomics is open to new findings, the challenge is the identification of compounds of significance and interpretation of affected biological pathways.^25^, ^26^

Some common methods used for indirect detection are the population‐wise discriminant approach and common fragmentation pathways.^21^ The population‐wise discriminant approach uses a comparison between treated individuals and a non‐treated population to identify markers of effect.^21^ An example of this approach for human disease research was a study conducted on 1211 subjects of whom 365 were patients with catecholamine‐producing tumours, known as pheochromocytoma and paraganglioma.^32^ Statistical comparison of the two patient groups showed a significant increase in dopamine and norepinephrine and significant decrease in epinephrine in patients with metastases. A biased non‐targeted screening can also identify compounds through mass‐defect filtering and common fragmentation pathways.^33^ Common fragmentation pathways aim to identify product ions which are shared between chemical families.^33^ This approach complements targeted with untargeted screening.

## 3 SAMPLE PREPARATION TECHNIQUES

Sample preparation has long been the minimalised and potentially compromised component of analytical method development. Moreover, sample preparation should be considered crucial to the experimental design for metabolomics since the subsequent elements of a workflow can only be as effective as the method used to extract the compounds of interest.^34^, ^35^ For metabolic studies, sample preparation methods should be as simple and universal as possible.^11^, ^19^, ^27^, ^36^, ^37^, ^38^ Sample preparation must consider multiple influences, which include protein concentration, analyte polarity and stability.^39^ Consistent sample preparation methods are essential for “omics” studies since physiological parameters such as diet, environmental effects and genetics will cause small changes and these may be misinterpreted if the sample preparation protocol introduces bias.^9^ Methods commonly employ steps to remove interfering compounds and thus reduce matrix effects.^10^, ^37^ Sample preparation needs to account for the collection containers used as they may release compounds which can interfere with the MS and/or NMR analysis.^27^ Some of the most common sample preparation techniques for MS‐based analysis methods are protein precipitation (PP), liquid–liquid extractions (LLE) and solid phase extraction (SPE).^40^, ^41^, ^42^, ^43^

### 3.1 Dilute‐and‐shoot

Dilute‐and‐shoot methods employ minimal sample preparation before analytical determination of the sample. It is commonly used for urine analysis^21^ but may suffer from matrix effects that result in ion suppression when compared with more comprehensive sample preparation methods.^44^

### 3.2 Protein precipitation

PP, similar to a dilute‐and‐shoot method for urine,^44^ is a rapid technique for blood plasma sample preparation.^19^, ^20^, ^45^ Protein content (approximately 35–40%) in blood needs to be removed to avoid issues with sensitivity and interferences during instrument analysis.^28^, ^38^ PP methods generally involve the use of a small volume of biological fluid (~100 μL) before quenching to preserve biological activity.^10^, ^12^, ^46^ Quenching is generally achieved through the addition of cold solvents, acids or rapid heating.^11^ Want et al. developed a PP method using methanol and found it to provide a large number of detected metabolites with less than 2% protein from serum.^47^ One issue with PP is ion suppression, which is particularly prevalent when using electrospray ionisation (ESI) in MS analysis. Ion suppression can be mitigated by reducing co‐extracted matrix interferences, improving chromatographic separation to avoid co‐elution of unknown compounds and by optimising the ionisation conditions for the MS‐interface.^48^

### 3.3 Solid phase extraction

SPE is a widely used sample preparation technique due to its high extraction yields and repeatability.^11^, ^49^ SPE works to isolate compounds by van der Waals interactions, dipole–dipole interactions, hydrogen bonding or electrostatic forces.^10^ Selectivity, via washing to remove matrix interferences and elution of desired analytes, is one of the main benefits of SPE.^11^, ^29^ However, this selectivity introduces bias by exclusion of compounds.^37^ To balance the need for selectivity with the desired compound scope, mixed‐mode sorbents show the most potential for implementation into metabolomic studies.^10^

### 3.4 Liquid–liquid extraction

LLE uses immiscible solvents to transfer target compounds between aqueous (i.e., hydrophilic) and organic (i.e., hydrophobic) phases.^50^ This technique allows for selection and isolation of target compounds with minimal matrix contamination.^50^ LLE has limited scope for affordable automation and may require large volumes of organic solvent.^28^, ^49^ LLE is often used in methods analysing tissue samples.^10^ Salting‐out can be used in conjunction with LLE to aid the recovery of organic compounds by increasing the ionic density of the aqueous phase.^51^ Purification of small organic molecules can be achieved with high polarity solvent mixtures used for extraction.^51^

### 3.5 QuEChERS

The Quick, Easy, Cheap, Effective, Rugged, Safe (QuEChERS) extraction is increasing in application for forensic studies using whole blood.^28^ QuEChERS is a two‐step extraction process that uses acetonitrile in the presence of a salt to extract analytes of interest followed by dispersive SPE for clean‐up.^52^ The implementation of QuEChERS can improve the extraction of both polar and non‐polar drugs in a range of matrices.^39^ Historically, QuEChERS has been frequently used in the pesticide and pharmaceutical industry, but it is gaining popularity within the metabolomics community.^53^

## 4 ANALYTICAL DETERMINATIONS

### 4.1 Gas chromatography–mass spectrometry (GC–MS)

GC–MS has excellent separation efficiency while maintaining reproducible retention times.^27^ Electron impact (EI) is the most commonly used ionisation technique for GC.^27^ The development of benchtop instrumentation during the 1970s and 1980s saw GC–MS become the gold standard for analytical determinations with increased sensitivity and specificity, together with the use of spectral libraries.^27^, ^29^, ^54^, ^55^ The major challenge for GC–MS is analysis of non‐volatile, highly polar and thermally unstable compounds.^55^ Compounds are often subjected to chemical modification,^55^ but these derivatised compounds can display limited stability.^27^ Recent development of “variable” or “soft” EI, using energies less than 70 eV, has the potential to increase the scope of GC–MS analysis for metabolomics.^56^ This can alleviate the complexities of traditional soft‐ionisation, such as chemical ionisation (CI), which require separate sources and hazardous reagent gases, where a laboratory does not have access to dedicated instrumentation. The application of GC–MS for metabolomics is particularly useful due to the availability of spectral libraries for easier identification of biomarkers.^57^

### 4.2 Liquid chromatography–mass spectrometry (LC–MS)

The 1990s saw the development of liquid chromatography (LC) in combination with MS to improve the ability to detect and characterise a broader range of analytes, particularly small polar compounds that are insufficiently volatile and/or too labile for GC‐MS.^54^ ESI is the most commonly used interface between LC and MS,^13^, ^49^ however Atmospheric Pressure Chemical Ionisation (APCI) may also be used for low molecular weight and non‐polar compounds.^58^ Tandem LC–MS (LC–MS/MS) methods were developed in the 2000s to provide the sensitivity and specificity for early metabolomic studies, complemented sometimes by NMR.^6^, ^36^, ^59^ At present, considerable effort is being made by instrument manufacturers to improve the use of LC–MS libraries, in part due to the expansion of metabolomics applications.

### 4.3 Liquid chromatography‐high resolution mass spectrometry (LC‐HRMS)

The evolution of analytical methods has seen improved sensitivity from microgram per millilitre (μg/mL) detection capabilities in the 1980s to nanogram per millilitre (ng/mL) in the 1990s then to picogram per millilitre (pg/mL) in the 2000s and 2010s.^29^ The mid‐2000s and then 2010s saw greater use of LC coupled to high resolution mass spectrometry (LC‐HRMS) technology for metabolomic studies.^10^, ^13^, ^21^, ^36^, ^55^, ^59^ Quadrupole time‐of‐flight (QTOF) and orbitrap instruments are increasingly popular due to their advantage of acquisition in full‐scan mode, high scanning speeds, accurate mass and high resolution.^15^, ^33^, ^59^ Full‐scan data allow for retrospective analysis of the presence of new compounds as they become known within the field, such as new psychoactive substances (NPS).^33^, ^55^ Several fields, such as environmental monitoring, food safety and forensic science, have demonstrated that LC‐HRMS allows for the screening and confirmation of a large scope of organic compounds.^33^

### 4.4 Hydrophilic interaction liquid chromatography (HILIC)

Reverse phase (RP) chromatography performs well for hydrophobic analytes, whilst a more polar approach, such as HILIC, is recommended for hydrophilic compounds which experience poor retention using RP chromatography.^11^, ^19^, ^60^, ^61^ Kouassi Nzoughet et al. reported the complementary metabolomics approach of using both RP and HILIC to monitor the effects of a trenbolone acetate/oestradiol implant administration where the results from both approaches agreed.^62^ HILIC stationary phases allow for a diverse separation mechanism and lower back pressure using an acetonitrile mobile phase.^61^ Buffered eluents not only maintain the pH of the mobile phase but also reduce electrostatic interactions.^61^, ^63^ Narduzzi et al. demonstrated the sensitive nature of a HILIC column in their comparison of ammonium acetate and ammonium fluoride where they assessed the column in terms of peak quality, intra‐day and inter‐day repeatability.^64^ The addition of ammonium fluoride proved to be optimal for all assessed parameters thus providing a better alternative for a mobile phase buffer for future HILIC studies. Despite these examples of its implementation, the use of HILIC columns has been scrutinised due to changes in sensitivity within small pH ranges, mobile phase variation and long re‐equilibration times.^65^ Therefore, alternatives, such as amino acid columns, have been gaining popularity in metabolomics.^66^

## 5 DATA PROCESSING AND STATISTICAL ANALYSIS

Quantitative bioanalysis involving data pre‐processing, normalisation, statistical tests and metabolite identification is well described by several groups^22^, ^23^, ^27^ and further discussed in this section.

Data pre‐processing may involve peak alignment, background subtraction and charge state evaluation.^10^, ^22^, ^23^, ^36^ Sample normalisation involves adjusting either the sample pre‐acquisition or the data signal post‐acquisition to ensure equal signals of different metabolites.^67^, ^68^ The ideal sample normalisation will result in a short distance between samples in the same biological group but a large distance between separate groups.^67^ While post‐acquisition normalisation is easier and often preferred due to the data size collected for metabolomic studies, pre‐acquisition can improve information on biological activities.^67^ Normalisation may also be applied to account for variations in different batches of data thus reducing systematic error.^68^ Centring is used to condense the data around zero rather than surrounding the mean of the metabolite concentrations.^23^ This allows for a reduction in variation to only that of significance in a metabolomics study. Scaling uses an adjustable factor to correct for differences in the fold change of the metabolites.^23^ Transformations, such as log and power functions, are also commonly used to make the distribution more symmetric.^23^ Normality (i.e., parametric behaviour) can be tested for through the use of distribution plots and significance tests.^69^ While there is often debate on the most appropriate statistical test to use, the Shapiro–Wilk test is generally agreed to be the most appropriate for normality testing among many researchers.^69^ It is favoured over the Kolmogorov–Smirnov test as it provides greater power.^69^

Statistical analysis of metabolomics data is commonly multivariate, although gene‐expression generally uses univariate analyses. Saccenti et al. summarise and review both univariate and multivariate analyses in relation to metabolomics in their review article.^70^ Univariate analyses investigate one variable at a time and commonly use *t* tests and analysis of variance (ANOVA). These statistical tests are corrected for, with methods like the Bonferroni and Benjamini‐Hochberg, to reduce the probability of false positives. Statistical analysis of metabolomic data generally involves a combination of supervised and unsupervised multivariate techniques.^69^ A supervised statistical tool requires both training and validation data sets to develop reliable models.^71^ The most universally applied unsupervised statistical tool for metabolomic studies is principal component analysis (PCA). This aids the visualisation of the data in a simplified manner to reveal underlying patterns and clusters. Hierarchical cluster analysis (HCA) is another unsupervised tool used to visualise similarities and differences within variables through a dendrogram.^72^ Commonly applied supervised methods include partial least squares (PLS), support vector machine (SVM) and artificial neural networks (ANN). PLS analyses independent variables to form a matrix containing dependent variables.^9^, ^11^, ^12^, ^67^ SVM classifies the data by finding the optimal hyperplane in an N‐dimensional space; where N is the number of features.^73^ ANN attempts to mimic the analysis and processing system of the human brain.^36^ These techniques develop models that enable the discovery of biomarkers following classification and the prediction of future data.^71^ One limitation of these techniques is the possibility of over‐fitting the data,^11^ which can lead to a loss in predictive power. However, this issue can be identified at the validation stage.

Pathway enrichment analysis is common for omics studies to identify compounds which are overrepresented.^12^, ^27^ Tools used for the enrichment of “omics” data allow for a better understanding of the metabolome and how biological systems influence it. This occurs through the reduction of complex data and increased interpretation.^13^, ^74^ Enrichment analysis may include over‐representation analysis (ORA), hypergeometric, Kolmogorov–Smirnov or Wilcoxon statistical tests.^74^

There are multiple issues pertaining to current data processing methods. One limitation of metabolomic analyses is that a “true” finding may not be considered significant in a statistical setting.^21^ Pre‐defined criteria set by the analyst for the statistical test applied may be too stringent to identify metabolites that are indicative of a change to the system.^75^ Therefore, Ortmayr et al. propose the use of fold change and its uncertainty as an alternative statistical assessment to avoid the exclusion of entities that may be suitable biomarkers.^75^ A lack of disclosure of the whole statistical workflow is another limitation or common error of the analyst.^27^ This issue is further highlighted by the poor harmonisation of metabolomics workflows, which require analysts to be proficient in a number of areas such as experimental design, sample preparation, analytical instrumentation and statistical analysis. Therefore, it is common to experience errors from the application of statistical tests that may make incorrect classifications leading to false‐negative results.^21^ Metabolomic studies use a range of software tools to analyse data, and this can lead to inconsistencies. Proprietary tools have the limitation of only working with a specific type of data defined by the vendor.^76^ These packages are usually “closed” systems with limited flexibility for the analyst to review the data pipeline. Software that is available on the open market is cost effective compared with the proprietary tools, but there can be resource implications for training and long‐term support, which is often provided by informal user networks.^77^

A major challenge for metabolomics is confirming the identification of putative metabolites when only a small amount of information is known about such compounds.^11^, ^20^, ^78^ Scalbert et al. provided a useful example for metabolite identification.^27^ Following initial information (e.g., [M + H]^+^, ^13^C isotopic pattern) for a putative compound being obtained,^78^ additional information (e.g., MS/MS, *in silico* analysis and spectral library comparison) can be performed to increase confidence about the identity. *In silico* fragmentation software, such as MetFrag, MS‐DIAL, Metlin and more, are often used to increase the annotation rate for putative biomarkers.^79^, ^80^, ^81^ Metabolome databases, such as the Kyoto Encyclopedia of Genes and Genomes (KEGG), PubChem, BioCyc/HumanCyc and the Human Metabolome Database (HMDB), are used to identify potential biomarkers.^74^ Confirmation can then be attempted by comparison with an authentic reference standard, if one is available.^11^ In the absence of a commercially available reference material, custom synthesis is required, but this is usually costly and results in considerable delays for confirming findings from metabolomic analyses. Another common limitation is the sample volume available for follow‐up analyses, which may require further procurement of incurred samples.

## 6 BEST PRACTICE AND PERFORMANCE STANDARDS

Notwithstanding the rapid expansion of metabolomics over the last 20 years, a consistent limitation is the extent to which workflows have been validated as fit‐for‐purpose. However, there is a considerable body of work to address this. The Standard Metabolic Reporting Structure (SMRS) group published recommendations for standardisation of experimental design and result recording.^82^ Similarly, Goodacre et al. proposed a framework for the standardisation of metabolomics studies.^83^ The diversity of “omics” workflows requires different approaches towards standardisation.^84^ This has evolved into the Metabolomics Standards Initiative (MSI).^85^ A challenge remains to find a balance between academia's desire for full disclosure and industry's need for protection of intellectual property.^86^ Moreover, it is essential that as the field continues to grow so do protocols surrounding sample collection and preparation, together with data analysis and interpretation.^86^, ^87^ Minimum requirements have been proposed for four areas; the source of biological samples, analytical methodologies, multivariate statistical methods and databases.^88^

Sample origin, storage and metadata (such as gender, age, weight and diet) related to the sample are essential.^11^, ^82^, ^89^ For sample collection and storage, it is important to consider freeze–thaw cycles and factors such as clotting time and temperature.^27^ Experimental design is a key part to attaining robust and reliable data from analytical methodologies used.^90^ When designing the experiment, samples should be replicated in a randomised order.^19^, ^82^ Important parameters to specify for analytical instruments are the manufacturer, model, software and settings used.^82^ For MS techniques, the instrument resolution, sensitivity, mass calibration and mass accuracy should be reported.^21^, ^82^

Quality control parameters of instrument stability, estimation of data reproducibility, reporting and exclusion of data should be documented.^19^, ^21^, ^82^, ^91^, ^92^ Depending on the analytical methodology chosen, suitable instrument calibration is essential for quality control purposes.^82^ Broadhurst et al. provided an in‐depth review on the harmonisation of metabolomics workflows with a particular focus on quality assurance and quality control.^93^ The review provides guidelines and recommendations into appropriate quality management protocols for maintaining system suitability and QC across the workflow. In particular, routine use of blank and pooled QC samples were emphasised together with reporting of the QC data within published work and through the use of databases.

The significance of the statistical modelling completed within research should also be a focus of future validation strategies. Currently, statistical modelling and validation is not consistently reported within the field. For univariate analyses, false discovery rates are a commonly encountered issue which is due to an inadequate sample size.^94^ This particular issue is notable when the number of variables outweighs the number of samples. However, this issue can be quite common for “omics” studies. Whilst the correlation and false discovery rate improve with a greater sample size, bias may also perpetuate.^94^ One way to avoid this is to align the metadata; for example, gender matching of different groups will aid in reducing bias. One harmonisation measure for future publications would be including all metadata related to the study to improve transparency. For univariate analysis, pure Bonferroni analysis was recommended by Broadhurst and Kell due to its ease of comprehension and implementation.^94^ One particular means of assessing statistical models is measurement of capability. The model's descriptive capacity is expressed as *R* ^2^, and the model's predictive power is defined as adjusted *R* ^2^.^95^ The distribution of the *R* ^2^ and adjusted *R* ^2^ values can give an indication into the statistical significance of the model.^95^ Moreover, PLS models can also be assessed by permutation test, classification accuracy, k‐fold cross‐validation, receiver operating characteristic (ROC) curves and area under the receiver operating curve (AUC).^96^, ^97^ For SVM models, common validation techniques of leave‐one‐out‐cross‐validation, n‐fold cross‐validation and split‐validation are employed to assess the model.^98^ An S‐plot, a proprietary model in the SIMCA software, determines the most relevant variables involved in the discrimination of the groups and/or samples.^99^ Variable importance in projection (VIP), available through open‐source software, measures the impact of each variable with a higher VIP score indicating an influential variable.^100^ Rubingh et al. demonstrate, through a study involving 50 obese and 50 lean patients, how having a small ratio between the number of subjects and variables can result in less trusted validation results.^101^ The study emphasised the need for a large cohort of subjects representative of the population when conducting tests that require cross validation in order to make suitable interpretations without portraying misleading information. The implementation of these measured capabilities within the field of metabolomics will promote harmonisation in determining the significance of statistical models. These statistical parameters can provide an indication into analytical bias and outliers within the data, thus allowing a determination of the validity of the model, with respect to biological variability.

Data formatting, such as naming conventions, should be harmonised and followed.^82^, ^85^ Data alignment and processing need to be harmonised to ensure errors are not introduced.^91^ It is common practice to normalise mass spectra to the most abundant (i.e., base) ion.^82^ Quality control measures should be considered for multivariate analysis in relation to how errors will be identified.^19^, ^27^, ^91^ The control sample population should be used for the comparison to the metabolic perturbations.^19^, ^82^ Ren et al. proposed suitable methodologies for statistical analysis in an attempt to assist analysts in the metabolomics field who have limited expertise in statistics.^71^ Considine et al. explained, in their review of metabolomics studies for biomarker discovery involving serum samples, that the reporting of data, such as data filtering, removal and processing, was often not clear or was incompletely reported.^92^ Therefore, the harmonisation of metabolomics studies still remains an area that requires improvement.

## 7 APPLICATIONS

### 7.1 Clinical

Metabolomics is used for insight into the complex regulatory processes of mammalian systems through metabolic variation.^102^, ^103^, ^104^ The majority of clinical applications has focused on studying advanced diseases with little focus on early onset diseases^102^ that would be beneficial for preventative medicine. For clinical purposes, drug screening allows for the improvement of patient care with treatment guidance.^55^ Human nutritional experiments have been a major focus for clinical application of metabolomics, particularly with the growing interest in gut microbiota as a good indicator of health, but other areas of interest include gastrointestinal disease, metabolic disease, cancer, neurological and psychiatric disorders.^102^

A thorough literature review by Yan et al. accentuates the need for the inclusion of cerebrospinal fluid (CSF) into clinical practice as a tool for detecting neuroinflammatory disorders in humans.^105^ It highlights a multitude of potential diagnostic biomarkers, such as the tryptophan‐kynurenine pathway, nitric oxide pathway, neopterin and lipid species, that enable differentiation between control and patient samples. Lai et al. developed and validated a HILIC‐ESI‐MS/MS method targeting the quantification of arginine, citrulline and ornithine, in relation to impaired nitric oxide synthesis, in human plasma.^106^ Four different methods, using a blank matrix, surrogate matrix, surrogate analyte and background subtraction, were investigated to establish a suitable quantitative method. Three of the four methods were successfully validated and applied to the analysis of 97 human plasma samples to measure the concentrations of the target analytes. The three validated methods showed negligible differences between the measured concentrations. Yan et al. also furthered previous research by developing an untargeted metabolomics method, using LC‐HRMS, for the analysis of CSF to identify diagnostic biomarkers of neuroinflammation.^107^ Statistical comparison, using orthogonal partial least squares‐discriminant analysis (OPLS‐DA), of a disease group of patients with acute encephalitis and an age‐matched control group revealed 35 metabolites able to discriminate the two groups. Nine metabolites originated from the tryptophan‐kynurenine pathway. Variation in the tryptophan‐kynurenine pathway, nitric oxide pathway and neopterin were indicative of neuroinflammation and thus can be implemented into clinical practice.

For routine clinical testing, urine is a common biological matrix due to its ease of collection.^55^ Urine allows for extended detection of both the parent drug and metabolites in comparison to blood.^55^ The sampling site for extraction of blood needs to be considered due to differences in arterial and venous sampling for the local release of compounds, such as catecholamines.^49^ A study by Michopoulos et al. investigated the use of dried blood spots as an alternative to plasma.^38^ This could make clinical testing, which can often be frequent for those with chronic conditions, less invasive. They found that dried blood spots were more concentrated than plasma due to the increased viscosity of blood, but the repeatability of the blood spots was not good in comparison. The PP plasma sample had the best repeatability. Nevertheless, this pilot study demonstrated the use of implementing dried biofluid spots for metabolomic analysis.^38^

Amino acids, lipids and hormones have previously been the focus of disease studies.^89^ Levodopa was first introduced as a treatment for Parkinson's disease 40 years ago and is still the preferred treatment.^45^ The blood–brain barrier (BBB) is not crossed by dopamine,^45^ and therefore, an alternative compound is needed for treatment. For example, a study investigating hypertension was conducted on 590 human volunteers.^108^ It was found through multivariate analysis that males had higher concentrations of metanephrine and methoxytyramine in their urine than females.

The increased incidence of chronic diseases is a challenge for the health field,^109^ and metabolomics could be a useful diagnostic tool for management. A targeted metabolomics approach, using 10 free organic acids was developed to profile hospitalised children's urine for metabolic or health disorders.^110^ The authors plan to expand the study to a larger set of organic acids in order to support other clinics in their diagnosis of these disorders.

### 7.2 Forensic toxicology

Forensic toxicology uses metabolomics to aid the identification of new psychoactive substances (NPS), which is a growing problem globally. Szeremeta et al. state that “metabolomics‐related procedures present an alternative strategy for the identification of biomarkers and might be highly beneficial to provide fast response to suspected NPS consumption and aid in the overall diagnostics of drug abuse or overdose.”^111^ Toxicologists are looking for a major change in the metabolome in response to the consumption of these drugs, and so, there are fewer issues associated with data extraction.^27^

The major question around drugs that are also present endogenously is whether they originated from the body (being naturally present) or from the bottle (an exogenous source). One particular endogenous compound, gamma‐hydroxybutyrate (GHB), is known to induce feelings of euphoria and to enhance sexuality, and therefore, it has gained popularity as a recreational drug and notoriety in drug‐facilitated sexual assault.^112^ Due to the rapid metabolism and small window of detection of GHB,^113^ metabolites of GHB have been proposed to extend detection windows with promising results using urine samples.^114^, ^115^ Hair testing is another method suggested for extending the window of detection due to the incorporation of drugs into this matrix. Recent progress in hair testing highlights the growing applicability of metabolomics to forensic testing.^116^, ^117^, ^118^, ^119^

Heroin and amphetamine‐type substances are potentially the most well‐known drugs in the wider community and therefore are a focus for forensic toxicology due to their recreational use. Potential heroin biomarkers were investigated in human plasma from 50 participants (20 heroin addicts with acute abstinence, 15 with prolonged abstinence and 15 controls) by Zhou et al. using ultraperformance LC–MS/MS.^120^ The major finding of the study was that alpha‐aminobutyric acid, alloisoleucine, ketoleucine and oxalic acid did not recover following the heroin administration. Plasma metabolites were found to experience severe change during the withdrawal period. Steuer et al. performed a similar metabolomics study investigating the administration of 3,4‐methylenedioxymethamphetamine (MDMA), amphetamine and mephedrone in human plasma using LC‐HRMS.^121^ It was found that energy metabolism, steroid biosynthesis and amino acids were the main groups affected by the different drugs with both increased and decreased concentrations. Specifically, linoleic acid and pregnenolone‐sulphate displayed similar alterations. These studies show the ability of metabolomics studies to advance the understanding of the pharmacology and metabolic mechanisms associated with the consumption of these drugs.

Adulteration of samples is an important consideration when conducting forensic toxicology analyses. Eisenbeiss et al. developed a metabolomics approach for the detection of adulterated hair samples.^122^ The authors found that the use of biomarker ratios allows for the discrimination of oxidative adulteration from unadulterated samples. Steuer et al. investigated oxidative adulteration of urine samples through a metabolomics approach.^123^ The ROC analysis revealed 5‐hydroxyisourate as the most suitable biomarker followed by uric acid. This study also highlighted the usefulness of an ERC as a reference point for the normalisation of a ratio or threshold.

### 7.3 Human sports anti‐doping

The proportion of doped athletes in a population of athletes at a specific time defines the prevalence of doping. This can be estimated using Bayesian networks to provide intelligence for authorities and their respective laboratories.^124^ Direct detection methods are then tailored to prohibited substances. It is due to a lack of influence from biological or genetic factors that they are considered to be sufficient proof of doping efforts.^21^ Narduzzi et al. reviewed untargeted metabolomics approaches to detect hormone doping in animals and then discuss its applicability for human athletes with a particular focus on a lack of application and validation of metabolomics methods.^21^ The review summarises the vast amount of known information about hormones and how they affect metabolism, indirect detection methods used in the animal and human fields, current limitations and expected effects on the metabolic system.

A particular focus of human anti‐doping is the use of anabolic androgenic steroids (AAS).^125^ A recent study by Raro et al. used two different analytical approaches, QTOF and Q Exactive both coupled to LC, to analyse urine collected pre‐ and post‐administration of testosterone cypionate.^126^ A “dilute‐and‐shoot” method was used to prevent analyte loss and samples were run in both positive and negative ionisation modes.^126^ Using the XCMS software and multivariate analysis, the biomarker, 1‐cyclopentenoylglycine, was identified and found in the results from both methods.^126^

Boccard et al. used targeted and untargeted metabolomics of urine samples to investigate steroid profiles following the oral administration of testosterone undecanoate.^127^ A series of supervised methods were applied, including N‐way‐partial least squares‐discriminant analysis (N‐PLS‐DA), O‐PLS‐DA and Shared and Unique Structures (SUS) plots, to identify metabolites of interest. Potential biomarkers were then confirmed using ROC curves inspection, with the results mainly being either glucuronide or sulphate steroid conjugates. Palermo et al. also used an untargeted workflow to analyse the urine steroidal profile following a testosterone administration study.^128^ This revealed significant metabolites related to circadian steroidal pathways and androgen metabolites which were both indicative of testosterone administration. This study provided a solid foundation for the consideration of external influences that cause variations in the metabolome.

Recombinant human growth hormone (rhGH) is a well‐known performance enhancing agent that regulates anabolism and lipolysis in humans.^129^ Misuse of rhGH is difficult to detect due to rapid turnover and inter‐individual variation from age, ethnicity and sex.^129^ Narduzzi et al. conducted an administration study using micro‐dosing techniques to investigate biomarkers indicative of growth hormone doping.^129^ Discriminant analysis using population‐wise modelling was able to distinguish between the control and treatment groups, but was subject to false positive results. Therefore, longitudinal modelling was used to account for variance within individuals thus allowing for more effective differentiation between the groups.^129^

Longitudinal profiling has been used in the human and equine fields through the Athlete Biological Passport (ABP) and the Equine Biological Passport (EBP).^30^, ^130^, ^131^ A longitudinal (i.e., intra‐individual) assessment refers to a series of tests completed over the course of time on the same individual.^131^ Metadata for potential covariances, such as gender, age and ethnicity, may be useful to improve the sensitivity of developed models by reducing intra‐individual variance.^124^, ^131^, ^132^ It is important to determine whether these parameters are time‐dependent for longitudinal assessments.^131^ Metabolomic principles have supported the expansion of the ABP to include a steroidal module and will likely do so for the planned endocrine module.^25^ Narduzzi et al. found through their investigation of rhGH administration that the leukopoietic, steroidal and endocrine biomarkers were able to correctly classify over 98% of samples. While the endocrine module of the ABP did not suffer false positives, it was limited in its classification individually with only 50% of treated samples being correctly classified due to the variable response to treatment in an athlete population. Therefore, the influence of covariance and external factors still needs to be considered and evaluated as they may have a large effect on the outcome.

### 7.4 Equine anti‐doping

Genetics, training and nutrition are all influencing factors, which determine how well a horse runs on the track.^133^ The horse has shown advanced aerobic and muscular capabilities that has been isolated through breeding.^12^ Common metabolomics studies of the equine athlete look at its exercise physiology, and therefore, metabolites are measured for energy production and utilisation.^12^, ^134^

Investigations into the health status of an equine athlete will provide information on biomarkers of disease and healthy athletes. Yuan et al. used an orthogonal ionisation approach to investigate the health status of the equine athlete when targeting compounds such as proteins, lipids and small polar metabolites.^134^ The one‐horse urine study revealed 46 proteins, 10 lipids and 474 small polar metabolites, which are indicative of a healthy mare. These findings can be used to track the health status of horses, specifically mares, and for future reference to other administration or health studies as a comparative measure. The expansion of this study to other horse genders would be beneficial to the field.

The study conducted by Le Moyec et al. showed that long endurance racing had a significant effect on plasma lipid and amino acid metabolite signatures.^135^ This research was furthered by Jang et al., who utilised OPLS‐DA along with variable importance plots and *t* tests as a statistical tool to analyse metabolic patterns before and after exercise in horses and predict 36 pathways.^136^ This study highlights the role of statistical analysis to aid metabolic discoveries by relating biomarkers to their metabolic pathways for routine drug testing and equine welfare. Also, biomarkers that will not be useful as doping indicators can be identified and excluded from further research. A Mach et al. pilot study showed promising results after a one‐horse study using metabolomics, transcriptomics and miR‐Nomics to predict racing performance.^137^ Kieken et al. studied urine and plasma following an administration of recombinant equine growth hormone (reGH) using an orthogonal metabolomics approach to detect metabolic differences between control and treatment groups.^138^ The OPLS models for both plasma and urine were assessed using descriptive (*R* ^2^(Y)) and predictive (adjusted *R* ^2^(Y)) capabilities to validate the discriminatory power of the proposed models. While there were no common ions of interest found between the two matrices, each still has a specific use, plasma being useful for unknown sample prediction and urine being useful for long‐term detection.

Recently, there has been a shift to utilise a metabolomics approach for the detection of endogenous compounds. As previously mentioned, the labile and variable nature of endogenous compounds makes them difficult to detect or establish a threshold for. Dopamine and related compounds are of particular interest due to the stimulant effects on the equine nervous system.^139^ It has been proposed that a lack of information on equine metabolism of dopamine‐related compounds may permit their abuse to go undetected in current screening efforts.^140^ Wynne et al. have done extensive research into dopaminergic manipulation and established a urinary threshold for 3‐methoxytyramine (3‐MT) of 4 μg/ml to combat misuse of compounds containing levodopa.^141^ This provided a good basis for further research into 3‐MT and potential dopamine‐related compound misuse. Stanley et al. found that tolcapone and its metabolites were readily detected in all samples for up to 18 h post‐administration and the dose administered in this study was thought to be a third or half of what would be used as a masking agent for dopamine‐related doping.^139^

Similar to the human field, steroid doping is a concern due to the difficulty in differentiating whether there was an exogenous or endogenous source.^142^ This was evident in a study conducted by Kaabia et al. where two matrices, equine plasma and urine, were used to develop a successful statistical model that enabled the extension of the detection period of nandrolone abuse in entire male horses.^143^ More intrinsic information was provided beyond the established threshold for nandrolone abuse. Chan et al. used an OPLS‐DA model to identify seven biomarkers that were indicative of steroidal aromatase inhibitor administration. From these, androst‐4‐ene‐3,6,17‐trione (6‐OXO) and androsta‐1,4,6‐triene‐3,17‐dione (ATD) extended the detection period to 4 and 9 days, respectively.^144^

Greater retrospectivity for the detection of prohibited substances is one of the goals of antidoping. This objective is especially important for equine antidoping as drug prohibition is enforced for performance enhancing and performance impairing substances. Equine serum and urine samples were analysed using a metabolomics approach following an administration study involving 11 horses that were given treatment with eye drops containing dexamethasone and prednisolone.^145^ Prednisolone was detected the day after administration was stopped, but dexamethasone was not. This study highlights the usefulness of establishing cut‐off values and clearance times through out‐of‐competition testing. Another study investigated the expansion of a detection window through the administration of the erythropoiesis stimulating agent, Mircera®, to three horses to study haematological and metabolic changes.^146^ Haematological studies revealed significantly elevated levels of haemoglobin and haematocrit. Statistical analysis, using an O‐PLS model, was able to differentiate pre‐ and post‐administration samples which extended the current detection window by 43 days. Metabolomics is therefore not only useful for the detection of analytically challenging compounds, but also the expansion of detection windows.

Duluard et al. conducted a longitudinal follow‐up study, focusing on detecting protein‐based drugs, recombinant human erythropoietin (rHuEPO) and reGH, on racehorses to investigate the applicability of metabolomics and transcriptomics as being an additional approach to current anti‐doping testing efforts.^30^ Using the 42 horses analysed for a 1‐year period, it was found that the OPLS predictive model was able to use 80 ions to differentiate between reGH‐treated horses and the control group. The study found the metabolomic profile of horses analysed throughout 2009 to be normal as they aligned with the non‐treated population.

### 7.5 Veterinary residues

Veterinary residues aim to detect the misuse of drugs in animals mainly for food safety purposes.^147^ The proposed use of metabolomics within the field would be to identify chemical residues within the animal sample,^148^ thus allowing for a determination of the impact in the cell metabolism that the contamination would have. Another approach, that is more focused on accreditation and regulation of food, would be identifying biomarkers relating to regulatory issues and compliance.^149^ Metabolomics also enables the determination of quality, taste, fragrance and more for the food product.^150^ The potential for improved application of metabolomics within the veterinary residues field was highlighted by many in review papers.^147^, ^150^, ^151^

Cacciatore et al. studied 10‐week‐old male and female veal calves, treated with a combination of 17β‐estradiol‐3‐benzoate, 19‐nortestosterone decanoate and dexamethasone, with the aim of detecting potential biomarkers for residue monitoring that were indicative of growth promoters.^152^ Within a 6‐week period of testing, it was found that the treated animals had an accelerated growth rate with the hormone treatment revealing a decreased level of immunoreactive inhibin in males, and the dexamethasone revealing a decreased level of osteocalcin. Therefore, both osteocalcin and immunoreactive inhibin were considered potential biomarkers for a screening assay to detect growth promoters. Courant et al. showed the use of an untargeted LC‐HRMS metabolomics method to detect the administration of clenbuterol in the urine of calves.^148^ Multivariate statistical analysis resulted in two different models tailored for the detection of clenbuterol administration, one during the treatment period and one for several days post‐administration. An OPLS‐DA model was able to identify ions of interest that were able to discriminate the two different testing periods.

Doué et al. used a similar approach to develop a metabolomics workflow for the investigation of growth hormone abuse in cattle where the target compounds were mostly proteins.^99^ The model was determined to be valid, using assessment factors such as *R^2^*, adjusted *R* ^2^ and cross‐validation, and successful in distinguishing the two sample classes: treated and control. The S‐plot revealed insulin‐like growth factor‐I, urea, non‐esterified fatty acid, insulin and cholesterol as the compounds with the most discriminating power.

Kouassi‐Nzoughet et al. used a LC‐HRMS metabolomics approach for the analysis of bovine serum to characterise the disruption of the metabolite profile when administered with trenbolone acetate and estradiol.^62^ A screening model was developed based on nine putative biomarkers, one of which was classified as dopamine based on elution time and MS/MS fragmentation. The model allowed for the discrimination of treated and control samples up to 4 weeks post‐administration. This research was furthered with a lipidomics approach using both HILIC and RPLC for optimal lipid coverage, to compare the effectiveness of both LC methods.^153^

Dervilly‐Pinel et al. highlight the need for guidelines covering the validation of untargeted methods.^149^ A metabolomics screening method was developed, for the detection of β‐agonist administration in calves, to showcase the validation criteria that should be presented in future studies of a similar nature. While the method was successfully validated, not all biomarkers used in the untargeted method were structurally elucidated which would be an important step to complete the untargeted metabolomics workflow.

## 8 CONCLUSION

Metabolomics continues to expand as a multidisciplinary field requiring expertise in biology, chemistry and statistical analysis. Workflow components such as experimental design, sample preparation, analytical determinations, data processing and statistical analysis, and validation strategies, were explored in this review. With greater scrutiny of interpretations made from metabolomics analyses in the applications presented, there will likely be an improvement in validation strategies. This would include the implementation of harmonised reporting criteria including information surrounding data processing. Another useful future direction would be the establishment of an equine metabolome database. While a significant proportion of the metabolome may be suspected to be similar to human, there are differences and it would be useful for researchers to have an easy access point for information on equine athletes. Expansion and integration of ABP modules and further development of the EBP would be useful to support an intelligence‐based approach to anti‐doping. Applications of metabolomics in the fields of clinical and forensic toxicology, and in the human and equine sports anti‐doping fields, were explored. This review highlights the potential of metabolomics into expanding current research efforts to identify biomarkers of interest and potentially indirect or unknown metabolite pathways.

## Data Availability

Data sharing is not applicable to this article as no new data were created or analyzed in this study.
